# Towards 3D basic theories of plant forms

**DOI:** 10.1038/s42003-022-03652-x

**Published:** 2022-07-14

**Authors:** Yi Lin, Juha Hyyppä

**Affiliations:** 1 School of Earth and Space Sciences, Peking University, Beijing, 100871 China; 2 Finnish Geospatial Research Institute, FI-02430 Masala, Finland

**Keywords:** Theoretical ecology, Plant ecology

## Abstract

Allometric, metabolic, and biomechanical theories are the critical foundations for scientifically deciphering plant forms. Their concrete laws, however, are found to deviate for plenty of plant specimens. This phenomenon has not been extensively studied, due to technical restrictions. This bottleneck now can be overcome by the state-of-the-art three-dimensional (3D) mapping technologies, such as fine-scale terrestrial laser scanning. On these grounds, we proposed to reexamine the basic theories regarding plant forms, and then, we case validated the feasibility of upgrading them into 3D modes. As an in-time enlightening of 3D revolutionizing the related basic subject, our theoretical prospect further sorted out the potential challenges as the cutting points for advancing its future exploration, which may enable 3D reconstruction of the basic theories of plant forms and even boost life science.

## Abstract (web summary)

In this Perspective, the authors discuss how state-of-the-art three-dimensional mapping technologies such as fine-scale terrestrial laser scanning can help us understand the theories of plant forms.

## Introduction: The established basic theories of plant forms show law deviations

In plant science, form, also termed appearance, shape, architecture, structure, or morphology, has long been followed with wide interest^1,2^—ranging from its measurement^3^ to its attribution^4^. For quantitative characterization and ecological analysis of its diversity and development, scientists have creatively developed multiple basic theories^5^. Allometric^6^, metabolic^7^, and biomechanical^8^ approaches can collectively outline the characteristics of plant forms. Such already-established basic theories have been able to generally shed light on the underlying patterns and reasons how and why plants show their distinctive forms^9^.

The allometric theory, alias the allometric scaling theory, can characterize the size relations of the body parts that constitute the integrative forms of plants^10–12^ and facilitate metering the different properties of plants in a relatively easy way. The metabolic theory, also termed the metabolic scaling theory, is aimed at quantitatively deriving the scaling of metabolic rate as a function of body sizes and environmental factors (e.g., in the scaling forms based on Cauchy’s theorems)^13–16^, in favour of learning the inner processes of plants through their forms. As for the biomechanical theory, it examines the forms and functions of plants in a mechanical way^17,18^ such as explaining the strategies used by plants for avoiding windbreaks, being self-supporting, and retaining their growth habits^19^.

These basic theories on the apparent structure, internal physiology, and external adaptability of plants, in principle, can elucidate their respective effects in modulating plants to grow into specific forms^6,20–22^. Flexible combinations of these theories have also been validated for explaining the multifold aspects of form-related characteristics for diverse plant species^23^. For generalizing and characterizing such functions, many “universal”-purposed models such as the Geometric Similarity model^24^, the West/Brown/Enquist (WBE) model^13^, and the Auto-Stress-regarded Bending model^25^ have been developed. Such models can act as bridges for probing why the anatomical and physiological scaling exponents of plants scale as quarter-powers of mass, such as the popular WBE model performing with the scaling exponent = 3/4 for gross photosynthetic rate, metabolic rate, and resource use^26^.

However, for many species with complex forms, specific laws under the frameworks of these basic theories have been found with various deviations, which may be caused by plant species differences^27^, plant self-shading^28^, crown ratio influencing^29^, or plant-plant interaction^30^. For example, scientists observed that the scaling exponent of 3/4 for the metabolic relationships is not common for some woody plant species^31^. Till now, it is still uncertain whether such law deviations are special phenomena or general modes for plants^32^. The reason is the lack of systematic studies focusing on this question^19^. This shortage is rooted in another technical limitation that is typically unavoidable in traditional plant measurements, namely, almost no efficient techniques for in-situ mapping the full structure of plants^33^.

## The state-of-the-art mapping technologies supply a new perspective

**Fig. 1:**
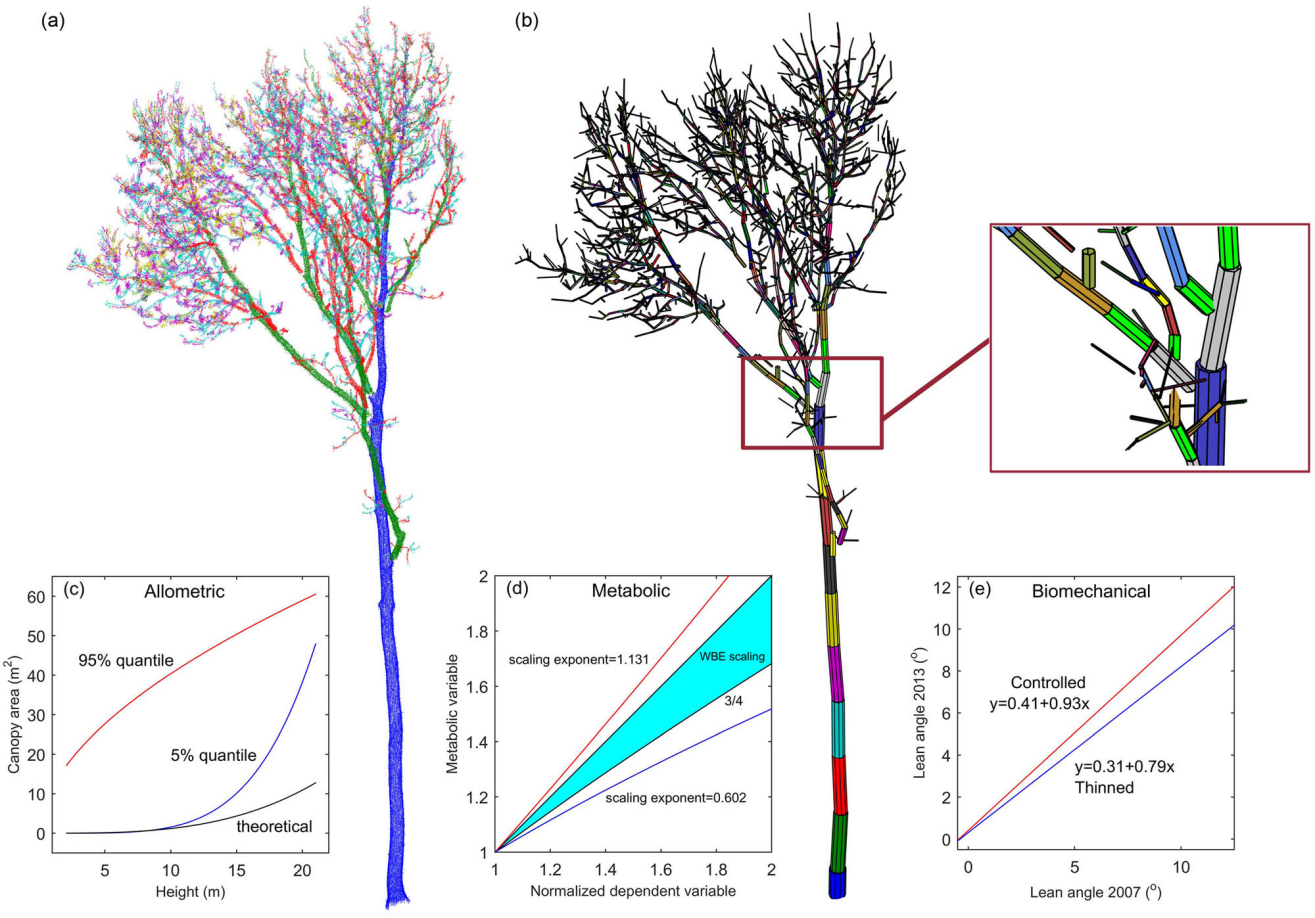
The background of inspiring a rethink of the basic theories of plant forms. Illustration of (**a**) the TLS-collected point cloud and (**b**) characterized 3D structure of a woody plant, with the high potential for boosting reexaminations of the deviations from its related (**c**) allometric scaling laws, (**d**) metabolic scaling laws, and (**e**) biomechanical laws. Note that (**a**) and (**b**) were generated based on published TLS data and Quantitative Structure Model software^34^, (**c**) was generated based on the published formulas of allometric scaling^44^, (**d**) was generated by following the published formulas of metabolic scaling, i.e., scaling exponent = 1.131 for gross primary productivity^45^, scaling exponent = 0.602 for gross photosynthesis ability^46^, and scaling exponent = 3/4 ~ 1 as derived from the adapted WBE model^47^, and (**e**) was generated in accordance to the published formulas of biomechanics (in terms of lean angle) before and after tree thinning^50^. The integration of the five images that reflect the kernel aspects of 3D reexamining the basic theories of plant forms draws the schematic framework to guide future studies.

The state-of-the-art mapping technologies such as terrestrial laser scanning (TLS) can survey and represent plant forms in a three-dimensional (3D) manner^34^. This is illustrated by the point cloud in Fig. 1a and the multi-cylinders-based branch modelling in Fig. 1b, respectively. This advantage can bring about a 3D revolution in how we look at trees^35^ and tree communities^36^. By extension, TLS can supply a new 3D perspective on the complex structures of plants and their communities. This reasoning is rooted in the fact that TLS has proved to be able to support deriving crown structural properties^37^, classifying species^38^, and estimating biomass^39^, which all serve as the foundations for a comprehensive understanding of plant forms. So far, these endeavours were mainly dedicated to bringing changes to the traditional plant phenotyping fields or providing supplementary points to the existing knowledge base^40–42^, not paying attention to the fundamental scientific questions that are explicitly involved in the basic theories of plant forms. Still, the scarce emergences of TLS-based attempts to gain further insight are inspiring, and we review these below.

For the allometric basic theory, a couple of studies based on TLS characterizing plants have detected its law deviations or even totally new laws in some specific scenarios. As a case study indicated, the conventional allometric equations are inadequate for estimating the biomass of a temperate species-mixed forest, while TLS-based tree stem modelling can serve as a potential solution of deriving non-destructive allometric equations for updating allometry and reducing the uncertainties in landscape-scale biomass estimations^43^. TLS was also used in an attempt to quantify the allometric variations of *Quercus mongolica* in semi-arid forests, and its derived allometric situation of low height but large canopy (as illustrated in Fig. 1c) matched just a low percentile of the derivations from the common Dynamic Global Vegetation Models (DGVMs)^44^. More such TLS-based studies exploring plant allometric modes and determinants would have implications for supporting from plant hydraulics analysis to DGVM improvement, and include 3D reexamining the allometric basic theory on plant forms.

In regard to the metabolic basic theory, some plans based on the TLS-mapped plant data have been proposed for exploring its new performance laws in special situations. As illustrated in Fig. 1d, the metabolic scaling laws of some plant species^45,46^ proved to show deviations from the simulations based on the WBE model^47^. To explain such deviations, it is needed to re-check the physiological assumptions regarding how the fractions and activities of the metabolically active tissues vary in organisms, and exploiting the key sources of this uncertainty relies on an accurate assessment of the surface areas of relatively smaller branches and twigs that make a disproportionate contribution to the total woody surface area^36^. TLS is applicable to finishing this basic task, and hence, it was supposed that TLS can help to test the existing allometric assumptions and give rise to new significant insights^36^. This hypothesis was verified by the finding that the TLS-measured scaling exponents of branch radius scaling ratio α and branch length scaling ratio *β* proved to diverge from the theoretical exponents of the WBE models^48^. Therefore, it was argued that TLS can be adopted as a useful tool for making comprehensive studies of plant biophysical processes and metabolic theories in ecology^49^, certainly covering the metabolic basic theory on plant forms.

As for the biomechanical basic theory, some endeavours based on the TLS-collected plant data have detected novel laws of biomechanical modes in some situations. As compared in Fig. 1e, the ecological effect of tree thinning can affect its biomechanical performance in terms of lean angle^50^. However, more comprehensive explorations of plant biomechanical properties, in terms of four typical biomechanical traits (such as two safety traits against wind and self-buckling, and two motricity traits involving how to sustain an upright position—tropic motion velocity and posture control)^19^, require more detailed information about plant forms. For this need, TLS has proved to be a potential solution for deriving plant structural features^38^, and such parameters can inspire more studies on plant biomechanical natures. A representative TLS-based biomechanical study proved to generate the answers to the specific questions on plant structure-wind ecological interactions, such as what decides critical wind speeds and whether trade-offs lie between such speeds and plant growth rates^51^. Such applicability of TLS can no doubt be extended to 3D reexamining the biomechanical basic theory on plant forms.

## 3D reexamining the basic theories of plant forms: allometric case study

**Fig. 2:**
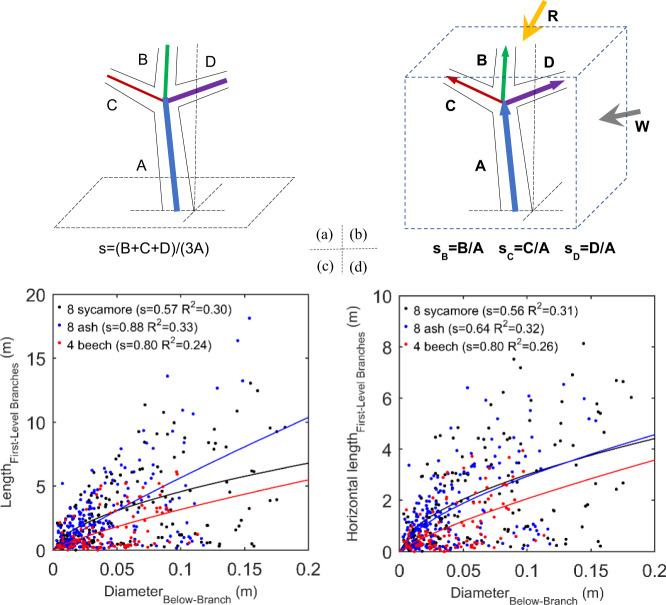
The 3D mode transition for reexamining the basic theories of plant forms. Illustration of the potential of proposing 3D allometric scaling for plants, via comparing, in principle, the effects of the (**a**) scalar—(length: A, B, C, and D) and (**b**) vector-parameter-based (length and orientation: A, B, C, and D) allometric scaling (s and s) characterizations that are based on the traditional and 3D-mode plant mapping technologies, respectively. The latter can open a new way for 3D upgrading the traditional allometric theory to adapt to the ecological analyses with the, in essence, vector-mode environmental forces such as solar radiance^55^ (R) and wind fields^56^ (W). Enabled by the proposed 3D theoretical framework, we found that the diameters of the trunk below branches are more correlated with the horizontal components of the first-level branches than themselves (**c**) vs. (**d**), for the samples of Sycamore (*Acer pseudoplatanus*, 180 first-level branches for all of its trees) and Beech (*Fagus sylvatica*, 104) tree species in the Wytham Woods, UK^52^, as characterized by a comparison of the performance in terms of *R*^2^.

As it is not sufficient to only propose the feasibility of 3D reexamining the basic theories of plant forms, we performed an allometric case study. Specifically, we proposed a new concept of 3D allometry instead of its traditional mode, as illustrated by the principle transition from Fig. 2a, b. The theoretical foundation of making such a proposal is that growth direction, in addition to the often used parameter of its magnitude, can better reflect the physiological, biomechanical, and ecological properties of plant organs, such as their orientation preferences in pressure response during their growths^50^. The definition of 3D allometry is that for a plant, its stem, branches, and leaves are characterized by introducing vector feature parameters, instead of scalar ones generally used under the traditional allometric theoretical framework. The scaling between such two vector parameters is valued by both the magnitude quantifying the comparative relation between their scalar components in any direction and the orientation information of that direction. The resulting 3D allometric theoretical framework can support breaking the aimed bottleneck—characterizing 3D plant forms and boosting their 3D allometric studies.

The validation of 3D allometry was conducted by running a case study of three woody plant species—eight Sycamore (*Acer pseudoplatanus*), eight Ash (*Fraxinus excelsior*), and four Beech (*Fagus sylvatica*, 104) trees in the Wytham Woods, UK^52^, with their 3D point clouds collected by fine-scale TLS. The allometric laws between their trunks and first-level branches were derived, and a novel finding emerged. Namely, for the specimens of Sycamore and Beech trees, the diameters of trunk below branches are more highly correlated with the lengths of the horizontal components of the first-level branches than themselves (Fig. 2c, d). Note that such an allometric analysis of the horizontal components of plant branches was almost impossible under the traditional allometric theoretical framework, testifying to the validity of our proposed concept. This finding, here regardless of its attribution, suggests the promising future of 3D allometry. In a theoretically broader sense, this world is intrinsically 3D, and thus, why not apply 3D allometry for plants?

The answer is clear. This conceptual proposal can inspire the community to develop new principles, models, and methods for characterizing and exploring 3D allometries in plants. The resulting solutions may more explicitly tackle the uncertainties such as the scaling exponents varying from less than 2/3 to larger than 1^32^ for different plant species and improve the situation regarding allometric scaling exponents with regard to plant species growing with complex forms^53^. As for how to 3D characterize such forms, this principle challenge in the traditional allometric theoretical system may no longer be a problem. In such circumstances, we can derive 3D allometric laws as the ground-truth data for validating the scientific inferences from plant biophysics^32^ to plant physiology^54^, since their accumulated ecological effects on plants, in the viewpoint of plant development^55^, result in the measured plant forms. In a nutshell, 3D allometry can stimulate the re-cognition of plant form-structural modes and re-thinking about allometry-based plant form-structural basic theories.

With regard to what and how environmental forces influence plant form-structural modes, the proposed 3D allometry can open a vector-feature-based ecological way of seeking the answers. As illustrated in Fig. 1b, the proposed vector feature parameters can adapt to the analyses of the ecological relationships between the growth forms of plants and the directional effects of their environmental forces such as solar radiances^55^ and wind fields^56^. Thus, plant structural ecology can be characterized with more orientation details, potentially solving the controversies arising from traditional explorations of the relationships between allometric trajectories and environmental stresses^57^. This potential may facilitate refining the ecological analyses of plant postures at the organ scale and refreshing the ecological inferences about plant structures at the individual to ecosystem levels. This advantage can help to break the bottleneck in bridging the gaps between the various scales of plant structural ecology^58^. In other words, the 3D allometric concept can project a 3D upgrading of plant structural ecology.

Beyond structural ecology, a key question is how plants adapt themselves to environmental forces to display their form-structural modes. The 3D allometric concept can pave the way for more comprehensively exploring the related form-structural-functional links. Under this 3D allometric principle framework, the pitfalls in traditional analyses of biomass allocation modes in plants^59^ can be addressed, and more specific 3D patterns of biomass allocations in branches of various inclination angles can be reflected. Such specified 3D allometric laws may mean that it is quite possible to quell the traditional allometry-derived debates^53^. Further, 3D allometry may bring breakthroughs to the often-assumed laws on plant form^54^, re-understand the basic theories on plant allometry^13^, and even open the totally new fields on plant structure. In all, the proposed 3D allometric concept may start 3D revolutions of plant form-structural-functional sciences in the future.

## 3D upgrading the basic theories of plant forms is of potential but challenging

This allometric case study suggested that 3D reexamining the basic theories of plant forms is of high potential for promoting their 3D upgrading and, thereby, disclosing more secrets about plant forms. This potential is valid not only for the three cardinal kinds of basic theories as considered in this study but also many other kinds aiming at the different aspects involving plant forms. Such aspects may include plant physiology^20^, biophysics^34^, biochemistry^15^, and biosystematics^5^. Their basic theories after 3D reexaminations will bring critical revolutions to the related fields as well, and this will be of considerable implications for from refreshing our knowledge on plant forms^2^ to updating the foundation for global ecosystem understanding^44^, even possibly projecting a new field.

The identified potential, however, does not mean that stepping forward from 3D reexamining to 3D theorizing is easy, as this process will be challenging. The challenges include more than the technical gaps^35,36^ between TLS data collections and the possible laws of the new 3D allometric, metabolic, and biomechanical basic theories. How to derive such potential laws may be the key challenges in this theoretical-level analysis. This can be mitigated by re-asking the questions that have been unclear under the traditional frameworks of the allometric, metabolic, and biomechanical basic theories of plant forms. For example, can biomechanical or optimal allocation theories better make accurate estimates of reproductive allometries for special plant species^60^? How is it possible to balance the weight of allometric scaling and resource limitation in predicting maximum heights and other features of plants^61^? Can the changes in plant structure allometry serve as the evidence of plant “liberations”^62^? To what degree can plant morphologic plasticity explain the law deviations from the metabolic scaling theory in special situations^63^. On the other hand, how can biomechanical performance constrain plant forms^64^? How can biomechanical design decide the long-term stability of plants in terms of the balance between weight increase and gravitropic reaction^25^? How can the common framework of structural mechanics account for the evolution of the whole geometry due to plant growth processes^65^? What can theoretically cause their diversities^40^? Collectively, the possible challenges may involve dealing with plant structural traits from the perspective of their forming mechanisms and coordinating the derived laws between the different kinds of new basic theories.

The challenges discussed above indicate that the existing basic theories of plant forms are far from being perfect, particularly for woody plant species that commonly grow with complex structures. However, the compilation of these challenges is useful for figuring out the potential cutting points for launching the future studies in this direction.

## Prospect: towards 3D basic theories of plant forms

**Fig. 3:**
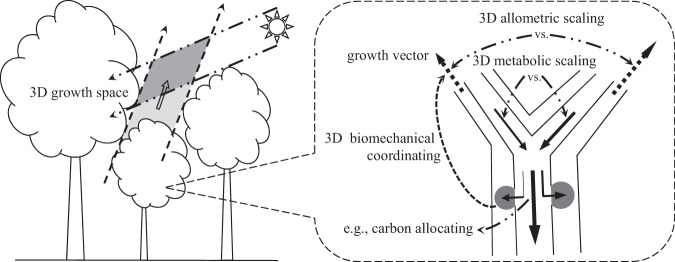
The principle feasibility of going towards 3D basic theories of plant forms. Illustration of the principle framework for supportively advancing 3D basic theories of plant forms, after finding their laws with deviations (Fig. 1) and proposing vector parameters for characterizing the form-related functional traits and their driving forces (Fig. 2). In terms of carbon that is the basic element of composing plant forms and the coherent inner scaling relationships between their allometric, metabolic, and biomechanical aspects^66^, exploring 3D growth space leads to branches growing in the form of vector, branches growing in the form of vector means 3D allometric scaling, 3D allometric scaling brings about 3D metabolic scaling, 3D metabolic scaling causes asymmetrical carbon allocating, asymmetrical carbon allocating gives rise to 3D biomechanical coordinating, and in the end back to where this cycle starts, 3D biomechanical coordinating alters growth vectors. Under this framework ranging from physical demand (left) to mechanism foundation (right), new 3D basic theories of plant forms can be comprehensively and systematically explored and developed.

To upgrade the basic theories of plant forms to 3D will ultimately lead to their 3D versions. This prospect is based on its viability in principle, as evidenced by Fig. 3, which illustrates its physical demand and mechanistic foundation. Substantially, developing 3D basic theories of plant forms is equivalent to returning to their 3D growth space. At the same time, the process of plant growth is in a 3D manner, ranging from carbon capture, carbon allocation, to carbon sequestration^66^. These physiological functions working within a 3D vascular structure engender 3D apparent patterns of metabolic scaling, biomechanical coordinating, and allometric scaling, integrally performing with coherent inner relationships. As specifically explained in Fig. 3, this facilitates systematically 3D reexamining and -reconstructing the basic theories of plant forms, even to the structure of plant collection^67^. The total picture in Fig. 3 provides an instructive framework for developing 3D basic theories of plant forms.

Overall, this study proposes the state-of-the-art in-situ 3D mapping technologies such as fine-scale TLS, for 3D reexamining the basic theories of plant forms. This work may promote more future explorations, particularly on plants with more diverse forms in the wild, towards 3D basic theories of plant forms. This trend may bring 3D critical changes and even breakthroughs to the scientific cognitions of plant forms and, in a broader sense, biological forms. All such merits will be of fundamental significance further for revealing more botanical and biological mechanisms for advancing life science from the bottom up.
